# Faculty of Medicine of Paris

**Published:** 1848-05

**Authors:** 


### FACULTY OF MEDICINE OF PARIS.

The provisional government of France, for reasons which, no doubt, will be given in due time, has removed the venerable Orfila from his office of Dean of the Faculty of Medicine of Paris, and appointed Bouillaud in his place.

